# SCORE Studies on the Impact of Drug Treatment on Morbidity due to *Schistosoma mansoni* and *Schistosoma haematobium* Infection

**DOI:** 10.4269/ajtmh.19-0830

**Published:** 2020-05-12

**Authors:** Charles H. King, Sue Binder, Ye Shen, Christopher C. Whalen, Carl H. Campbell, Ryan E. Wiegand, Annette Olsen, William Evan Secor, Susan P. Montgomery, Rosemary Musuva, Pauline N. M. Mwinzi, Pascal Magnussen, Safari Kinung’hi, Gisele N. Andrade, Amara E. Ezeamama, Daniel G. Colley

**Affiliations:** 1 Center for Global Health and Diseases, Case Western Reserve University, Cleveland, Ohio;; 2 Schistosomiasis Consortium for Operational Research and Evaluation, Center for Tropical and Emerging Global Diseases, University of Georgia, Athens, Georgia;; 3 Department of Epidemiology and Biostatistics, University of Georgia, Athens, Georgia;; 4 Division of Parasitic Diseases and Malaria, Parasitic Diseases Branch, Centers for Disease Control and Prevention, Atlanta, Georgia;; 5 Swiss Tropical and Public Health Institute, Basel, Switzerland;; 6 University of Basel, Basel, Switzerland;; 7 Section for Parasitology and Aquatic Pathobiology, Faculty of Health and Medical Sciences, University of Copenhagen, Copenhagen, Denmark;; 8 Centre for Global Health Research, Kenya Medical Research Institute, Kisumu, Kenya;; 9 Centre for Medical Parasitology, Faculty of Health and Medical Sciences, University of Copenhagen, Copenhagen, Denmark;; 10 National Institute for Medical Research, Mwanza Research Centre, Mwanza, Tanzania;; 11 Escola de Enfermagem, Universidade Federal de Minas Gerais, Belo Horizonte, Brazil;; 12 Department of Psychiatry, College of Osteopathic Medicine, Michigan State University, East Lansing, Michigan;; 13 Department of Microbiology, University of Georgia, Athens, Georgia

## Abstract

The Schistosomiasis Consortium for Operational Research (SCORE) was funded in 2008 to improve the evidence base for control and elimination of schistosomiasis—better understanding of the systemic morbidities experienced by children in schistosomiasis-endemic areas and the response of these morbidities to treatment, being essential for updating WHO guidelines for mass drug administration (MDA) in endemic areas. This article summarizes the SCORE studies that aimed to gauge the impact of MDA-based treatment on schistosomiasis-related morbidities. Morbidity cohort studies were embedded in the SCORE’s larger field studies of gaining control of schistosomiasis in Kenya and Tanzania. Following MDA, cohort children had less undernutrition, less portal vein dilation, and increased quality of life in Year 5 compared with baseline. We also conducted a pilot study of the Behavioral Assessment System for Children (BASC-2) in conjunction with the Kenya gaining control study, which demonstrated beneficial effects of treatment on classroom behavior. In addition, the SCORE’s Rapid Answers Project performed systematic reviews of previously available data, providing two meta-analyses related to morbidity. The first documented children’s infection-related deficits in school attendance and achievement and in formal tests of learning and memory. The second showed that greater reductions in egg output following drug treatment correlates significantly with reduced odds of most morbidities. Overall, these SCORE morbidity studies provided convincing evidence to support the use of MDA to improve the health of school-aged children in endemic areas. However, study findings also support the need to use enhanced metrics to fully assess and better control schistosomiasis-associated morbidity.

## OVERVIEW

The Schistosomiasis Consortium for Operational Research (SCORE) was funded in 2008 to improve the evidence base for control and elimination of schistosomiasis.^1^ As part of its mission, the SCORE sought to develop a better understanding of the anatomic and systemic functional morbidities experienced by people at risk in schistosomiasis-endemic areas and their response to antischistosomal praziquantel treatment. Such data are essential for updating WHO guidelines for schistosomiasis control and making the case for mass drug administration (MDA) in endemic areas. This article reviews and summarizes the results of the SCORE-supported projects that detailed specific human health impacts of *Schistosoma* infection and their response to treatment.

People living in schistosomiasis-endemic areas may spend one-third to a half of their lives carrying *Schistosoma* parasitic worms because their continuing environmental exposure leads to overlapping schistosome infections.^2^ Morbidity associated with schistosomiasis is caused by parasite eggs that are deposited daily into the human host’s organs, creating thousands of foci of granulomatous inflammation, particularly in the bowel and liver (*Schistosoma mansoni*, *Schistosoma mekongi*, and *Schistosoma japonicum*) or urogenital organs (*Schistosoma haematobium*). Although these granulomas can cause critical dysfunction of the affected organs, more frequently, the granuloma’s chronic inflammation contributes to multiple systemic deficits, including chronic pain,^3,4^ diarrhea,^3^ fatigue,^5,6^ reduced quality of life,^7,8^ anemia of chronic inflammation,^9^ impaired childhood growth and development,^10,11^ blunted response to childhood vaccines,^12,13^ and impaired school performance.^14,15^ The disease impact can become permanent, persisting even after the active infection has resolved.^16^ Often overlooked as part of *S. haematobium–*associated morbidity, genital schistosomiasis with pain, bleeding,^17^ and subfertility^18^ can result in increased rates of HIV transmission.^19^

Current WHO strategies for schistosomiasis morbidity control focus on regular delivery of antischistosomal drugs to at-risk populations as a form of “preventive chemotherapy” to limit (or even reverse) the impact of active infection and to prevent new disease sequelae from developing.^20,21^ Prioritization for control of neglected tropical diseases such as schistosomiasis is often carried out using disability-adjusted life years (DALYs) within international governmental and nongovernmental programs.^22,23^ The DALY construct is based on the perceived disability and persistence of a condition and is used to rank the relative contribution of different disabling conditions to the overall global burden of disease.^21,24^ The DALY rankings are sometimes assumed to reflect the importance of a given disease to world health.^25^ The DALYs attributed to schistosomiasis are derived primarily on the prevalence of classic organ-specific chronic anatomic pathologies of schistosomiasis (hepatosplenic disease and bladder fibrosis) observed in the most severe chronic infections, and not on the systemic functional conditions that are also related to *Schistosoma* infection. In response, SCORE researchers decided to use an expanded set of metrics for morbidity appraisals during the course of their MDA trials. These included nutrition and growth assessments, anemia testing, exercise capacity, and formal measurement of school behavior and of health-related quality of life before and after treatment.^26^ The impact of disease-associated stigma and depression,^27^ especially related to female and male genital schistosomiasis, is undoubtedly part of the disabling impact of schistosomiasis. Unfortunately, SCORE resources did not allow for study of these latter effects nor was there a sufficient timeline to evaluate the link between *Schistosoma* infection and long-term focal and systemic pathologies that persist beyond the period of active infection.^28^

Underlying all SCORE projects was the goal of providing data that would provide evidence to help program managers make decisions related to controlling and eliminating schistosomiasis. A related issue was providing evidence that would convince ministers in endemic countries to prioritize schistosomiasis treatment and prevention. Given the underestimation of DALYs related to schistosomiasis, in part related to the lack of high-quality data on the impact of lower intensity infections and the lifetime consequences, the SCORE saw the need to revisit and contribute data to help redefine schistosomiasis “morbidity” based on newer developments in the field. Its specific aim was to reevaluate how regular MDA could improve the health of school-aged children in schistosomiasis-endemic areas.^26^ As a result, the SCORE portfolio related to morbidity and its control included separate longitudinal cohort studies, a school behavioral assessment study, and two systematic reviews and meta-analyses of previously published data on infection-related morbidity outcomes.

## SCHISTOSOMIASIS CONSORTIUM FOR OPERATIONAL RESEARCH LONGITUDINAL COHORT STUDIES

Because there were few clinical research studies evaluating the long-term benefits of praziquantel in terms of prevention of new disease or amelioration of existing disease, SCORE partners incorporated nested comparison studies of the impact of MDA on *Schistosoma* infection–associated morbidity in school-aged children in each of the SCORE prospective randomized gaining control studies.^26^

The gaining control studies were large, cluster-randomized studies, with communities randomized to receive either two or four MDAs during a 4-year intervention period, with a follow-up assessment in Year 5.^29^ The morbidity cohort studies discussed here were conducted in the gaining control studies in Kenya and Tanzania and included four to six communities from the study arm receiving the most intensive treatment (4 years of MDA through community-wide treatment [CWT]) compared with four communities from an arm receiving standard every-other-year MDA through school-based treatment (SBT).^26^ Morbidity studies were also initiated in the gaining control studies in Niger and Mozambique, but these were discontinued after baseline data were collected because of a failure to appropriately randomize communities in Niger (resulting in dramatically different starting prevalences among study arms) and to extremely high loss to follow-up rates in Mozambique related to very low school attendance there.

One hundred 7- to 8-year-old children per community were targeted for enrollment at baseline (Year 1) for the cohort studies, with a goal of having 800 children prospectively monitored in each country.^26^ The decision to focus on this age-group was based on their greater likelihood of availability for follow-up throughout the 5-year study period.

After considering 17 different types of morbidity-related markers, the outcomes chosen for measurement in the planned 5-year longitudinal cohort studies were as follows:^26^

1. anthropometric measures: age-standardized height, weight, body mass index, mid-upper arm circumference^10,30,31^;
2. blood hemoglobin^3,9,32,33^;
3. exercise tolerance measured by the 20-m shuttle run (beep) test^34–36^;
4. health-related quality of life, measured by the standardized Pediatric Quality of Life (PedsQL) survey instrument^7,37^; and
5. ultrasonography of the abdomen and liver (for *S. mansoni*) or of the kidneys and bladder (for *S. haematobium*), using standardized WHO protocols.^38,39^

At baseline, 62% of children in the selected *S. mansoni* communities in Kenya and Tanzania had detectable eggs in their stool and 10% had heavy infections (≥ 400 eggs/g feces). Heavy *S. mansoni* infections were associated with increased baseline prevalence of anemia, although children with moderate or heavy intensity infections had lower odds of having physical wasting.

Prevalence of egg-positive infection in the combined *S. haematobium* communities in Mozambique and Niger was 27%, with 5% of individuals having heavy infection (≥ 50 eggs/10 mL urine). In contrast to the findings at the *S. mansoni* study sites, at baseline, it was light intensity *S. haematobium* infections that were significantly associated with anemia. They were also associated with lower scores in the social domain of the standardized PedsQL inventory survey. Individual country-level baseline findings for Kenya and Tanzania have been published elsewhere,^8,40^ and the baseline results from the four countries that initiated subtle morbidity studies have been summarized by country in tabular form by Shen and others.^26^

The Kenya and Tanzania gaining control studies were able to successfully complete their Year 3 and Year 5 cohort reexaminations.^41–43^ In a secondary analysis of their pooled *S. mansoni* longitudinal data, overall infection intensity and odds of infection were significantly reduced in both treatment arms of the study.^44^ Furthermore, both annual CWT and every-other-year SBT were associated with reduced odds of undernutrition in Year 5 (at the time, the children were aged 12–13 years). They also had reduced odds of portal vein dilation on ultrasound, as compared with their baseline when they were 7 or 8 years old. Although the prevalence of anemia did not improve significantly, this may have been confounded by increased malaria prevalence in the years following baseline examination in Kenya; malaria was not assessed in Tanzania. For the combined Kenya and Tanzania cohorts, growth stunting worsened in the areas receiving biennial SBT and VO_2max_ scores declined under both CWT and SBT regimens.

After adjusting for imbalance in starting prevalence between the two study arms,^44,45^ children in communities receiving annual CWT were found to have had significantly greater reductions in infection prevalence and intensity than those in communities receiving only biennial SBT. Although health-related quality-of-life scores improved in both study arms, children in the CWT communities gained significantly more. In aggregate, these SCORE findings suggested that programs implementing annual CWT are likely to achieve better overall *S. mansoni* morbidity control than those implementing biennial SBT alone.^44^

## SCHOOL BEHAVIORAL ASSESSMENT STUDY

There has been ongoing controversy about the public health benefits of “deworming” via MDA and whether regular treatments of school-aged children can improve their school performance and contribute to greater educational achievement.^46^ The SCORE investigators in Kenya attempted to rigorously investigate this issue by using a validated multidimensional survey instrument, the Behavioral Assessment System for Children (BASC-2), to assess emotional and behavioral problems among children in grades two through six.^15^ Thirty-six children in six schools within *S. mansoni*–endemic communities near Lake Victoria were assessed both parasitologically and with the BASC-2 Teacher Rating Scale, both before and 3 weeks after MDA delivery. Teachers were blinded to the infection status of these participating children. Following this initial assessment of behavior using the BASC-2, study children were given praziquantel by MDA using a standard SBT approach, along with all other children in their schools. Then, the BASC-2 was repeated for the same children who had completed the pre-MDA BASC-2 assessment.

Participating children’s BASC-2 scores improved significantly after treatment in each of the “problem” behavioral categories, with fewer externalizing problems (hyperactivity, aggression, and conduct problems that are disruptive in nature), internalizing problems (anxiety, depression, somatization, atypicality, and withdrawal), and school problems (academic difficulties, attention problems, and learning problems).^15^ Changes in BASC scores were seen in both egg-positive and egg-negative children.

A high level of heterogeneity in behavior score changes was observed among children who were initially egg negative, with posttreatment scores not changing much for some egg-negative children but changing dramatically for others. This could reflect the fact that because of its limited diagnostic sensitivity, a negative Kato–Katz stool examination in an *S. mansoni*–endemic zone does not exclude the presence of symptomatic low-level chronic infection.^47–50^ Because such low intensity infections often go undetected by stool testing, our posttreatment behavioral findings among egg-negative children suggests that their praziquantel MDA likely had a treatment-specific impact on their performance. It supports the concept that MDA can improve school performance and have a greater effect in endemic areas than that measured by only focusing on treatment impacts for *S. mansoni* egg-positive children.

## META-ANALYSIS OF COGNITION AND SCHOOL PERFORMANCE OUTCOMES

The SCORE’s Rapid Answers Project initiative^51^ used systematic reviews and meta-analysis of available data to answer other policy-relevant questions about schistosomiasis morbidity control. Two of these projects involved assessment of the impact of active infection and its drug therapy on risk of morbidity due to *Schistosoma* infections.^14,38^ Brief graphical summaries of their findings are provided in Supplemental information File S1 and File S2.

First, in developing a meta-analysis of the effects of infection and treatment on children’s cognition and school performance, we performed a systematic review of the available published data before 2016.^14^ Although there was a relatively large body of published reports and studies on childhood cognitive development and school performance, the variability in study quality necessitated a critical synthesis of their aggregate findings. A SCORE-affiliated team headed by Dr. Amara E. Ezeamama performed a systematic review of more than 2,900 articles and produced a meta-analysis of 30 relevant studies.^14^

Our findings from the meta-analysis indicated that compared with uninfected children or with children who received praziquantel treatment, children who had active *Schistosoma* infection or who had not been treated had significant deficits in school attendance and scholastic achievement and deficits in formal tests of learning and memory.^14^ By contrast, there were no significant differences between the two groups in tests of reaction time or of innate intelligence. *Schistosoma* infection–related deficits in learning and memory tests, but not scholastic achievement, were invariant to study design across the range of observational and interventional studies included in the meta-analysis.

## META-ANALYSIS OF DRUG-MEDIATED REDUCTION IN *SCHISTOSOMA* INFECTION INTENSITY AND ITS EFFECTS ON INFECTION-ASSOCIATED MORBIDITY

Praziquantel treatment is not completely curative for *Schistosoma* infections, particularly when heavy infection is present.^52^ Furthermore, praziquantel does not protect against rapid reinfection of those who live in high-risk environments.^53,54^ However, most individuals do experience a considerable reduction in the intensity of their *Schistosoma* infections after treatment, which has often been assumed to be a proxy for decreased overall morbidity.

Our second morbidity-related meta-analysis evaluated the actual scale of morbidity reduction when treatment reduces intensity of infection, even in the absence of cure.^38^ The SCORE also wished to determine whether there is a way to translate commonly reported “egg reduction ratios” into a quantifiable morbidity reduction benefit. The aim was to provide a conversion factor to capture “morbidity control” for later cost-effectiveness studies that would compare different schistosomiasis control strategies.^55^

The SCORE partners, led by Dr. Gisele N. Andrade, conducted a systematic review of 309 studies that recorded morbidity levels in affected individuals or populations before and after drug treatment interventions.^38^ A technique called meta-regression was performed to correlate the observed reductions in parasite egg outputs with the posttreatment reductions in measured morbidity prevalence.

The study found that larger reductions in measured egg output following treatment were indeed correlated with lower odds of continuing to have most kinds of infection-associated morbidity. Specifically, for *S. mansoni* and *S. japonicum*, more profound reductions in egg output were linked with less liver morbidity, and for *S. haematobium*, better treatment response in terms of egg output was linked to reduced odds of urinary tract bleeding and deformity.^38^ Thus, despite posttreatment persistence of active infections, the meta-analysis results suggested that even if total elimination of egg output is not achievable in all patients within endemic areas, MDA can still result in significant reductions in *Schistosoma*-associated morbidity prevalence if average reductions of egg output of ≥ 90% are achieved.

## SUMMARY AND NEXT STEPS

There is a need to better define the impact of *Schistosoma* infection in early childhood and its effects on concurrent and cumulative scholastic achievement.^56^ Such loss of human capital may actually represent the bulk of schistosomiasis-related disability within endemic areas.^57^

The SCORE morbidity studies, by adding new markers for physical and psychological functioning, provided better focus on the impact of *Schistosoma* infection on human performance. However, the studies’ scope was still limited by available resources, and in some cases, the effects of treatment in reducing morbidity were smaller than expected. Now, in follow-up to our assessments of functional morbidities, we think that larger cohort studies, involving different age-groups, additional testing, and tracking of coinfections, diet, and other effect modifiers are clearly needed.^58^ In follow-up to the SCORE BASC-2 behavior study,^15^ more extensive and more detailed psychological and behavioral studies will help triangulate and refine estimates of the overall impact of *Schistosoma* infection on childhood scholastic performance.

The analyses presented here reinforce the need for a complete reassessment of DALYs related to schistosomiasis. The number of morbidities included in DALY disability weighting needs to be expanded, and the often subcurative effect of praziquantel needs to be considered in case count estimation. One of the approaches buttressing the calculation of DALYs in the Global Burden of Disease program is the “counterfactual,” that is, what global burden would be like if the disease came under control or were eliminated?^59^ Current DALY estimates based on *Schistosoma* infection intensity assume that the prevalence of high-intensity infections is declining in the face of MDA implementation. This is not always true,^60^ and we suggest that newer estimates of persistent posttreatment infection be included in DALY calculations for schistosomiasis. These changes would provide a more realistic valuation of the schistosomiasis-associated DALYs that can be averted through MDA intervention.

Current WHO targets for control are primarily focused on reducing infection intensity.^21^ There is a clear need to redefine the required community-level prevalence targets for effective “morbidity control.” Large-scale initiatives such as the Morbidity Operational Research for Bilharziasis Implementation Decisions (MORBID) study^61^ are being planned to more clearly demonstrate the level of post-MDA community infection prevalence and intensity that are associated with persistent disease, in terms of both anatomical and functional pathology. The use of newer more sensitive point-of-care rapid diagnostics^62^ for both infection and morbidity will facilitate this assessment. Moving beyond the use of egg-counting diagnostics, which underestimate the prevalence of low-intensity infections,^50,63–66^ should enhance our ability to determine the attributable role of *Schistosoma* infections in widely prevalent multicausal pathologies such as anemia, growth stunting, cognitive dysfunction, depression, and infertility. This expanded knowledge will be critical for accurate estimation of the actual worldwide disease burden of *Schistosoma* infections and for optimal decision-making regarding health policy investment and development.^58^

## Supplemental files

Supplemental materials
