# Supplementary material for: SCORE Studies on the Impact of Drug Treatment on Morbidity due to Schistosoma mansoni and Schistosoma haematobium Infection
Source: Am J Trop Med Hyg. 2020 May 12;103(1 Suppl):30–5. doi: 10.4269/ajtmh.19-0830 (PMC7351303; doi:10.4269/ajtmh.19-0830)
Supplement: Supplementary file 1 [file tpmd190830.SD1.pdf]

SCORE Rapid Answers Project (RAP):

Cognitive Deficits and Educational Loss in Children with Schistosome Infections

Summary: Empirical evidence for cognitive or education benefits of treating *Schistosoma* infection in children is limited. In a study completed in 2016, we addressed this knowledge gap by synthesizing information from 30 relevant epidemiologic studies reporting on 38,992 children between 5-19 years of age from 14 countries. In those studies, children with *Schistosoma* infection, or those who had not received treatment, were compared to unifected children or to children dewormed with praziquantel. Children with *Schistosoma* infection or who had not been de-wormed performed worse on psychometric tests of learning and memory. However, they performed similarly to the uninfected or dewormed children in tests of innate intelligence or reaction time. Infected or non-dewormed children had less school attendance and poorer scholastic achievement. Overall, the presence of *Schistosoma* infection or non-dewormed status was associated with educational, learning, and memory deficits in school-aged children. The combined evidence suggests that early treatment of children in *Schistosoma*-endemic regions could mitigate these deficits.[1]

Questions:

1. Among school-aged children examined in the context of cross sectional or case-control studies, is *Schistosoma* infection associated with worse performance in neurocognitive tests, or with educational loss?

2. Among school-aged children enrolled in prospective studies with specific treatment for *Schistosoma* infection, is lack of treatment with praziquantel associated with worse performance in neurocognitive tests, or with educational loss?

Key Finding:

Schistosoma infection/non-treatment was significantly associated with educational, learning, and memory deficits in school-aged children.

Pooled standardized mean difference (SMD) estimates of *Schistosoma* infection/non-treatment effects on educational/cognitive loss – with evaluation of study heterogeneity and publication bias

	# Studies	SMD (95%CI)[1]	Heterogeneity[Ψ] I^2	Publication bias P-value[α]
**Cognitive Domain**				
Memory	8	**-0.28 (-0.52, -0.04)**	78.6	0.786
Learning	6	**-0.39 (-0.70, -0.09)**	79.4	0.793
Intelligence Quotient Based Assessments	4	-0.25 (-0.57, 0.06)	74.8	0.450
Reaction Time	6	-0.06 (-0.42, 0.30)	88.5	0.142
**Educational Loss Assessments**				
Achievement	16	**-0.58 (-0.96, -0.20)**	97.9	0.595
School Attendance	16	**-0.36 (-0.60, -0.12)**	98.7	0.991

[1]Standardized Mean Difference (SMD) < 0 suggests a negative effect of infection/non-treatment on the indicated outcome; SMD > 0 indicates a positive effect of infection on respective outcomes. Bold font indicates statistically significant differences.
Ψ: measures the extent to which there is heterogeneity across studies in terms of underlying results.
α: evaluates the tendency for increased publication of studies that show a statistically robust finding; a P < 0.05 suggests presence of publication bias.

The findings of infection-related cognitive deficits and educational loss reported here are clinically relevant and should become an essential pillar in the design of schistosomiasis-related health policy. They reinforce the need to treat children with schistosomiasis early in life, so as to reduce their cumulative cognitive and functional morbidities.

Schistosomiasis Consortium for Operational Research and Evaluation
CTEGD, University of Georgia, Athens, GA 30602
phone: 706-542-3334 • fax: 706-542-3582 • http://score.uga.edu

Cognitive Deficits and Educational Loss

Examples of significant impact of *Schistosoma* infection - the gray boxes show estimates of SMDs and their 95% confidence intervals for individual studies that assessed learning and memory. Based on our meta-analysis, the blue diamonds indicate the overall summary estimates of the respective impacts of *Schistosoma* infection on measured learning and memory scores among affected children.

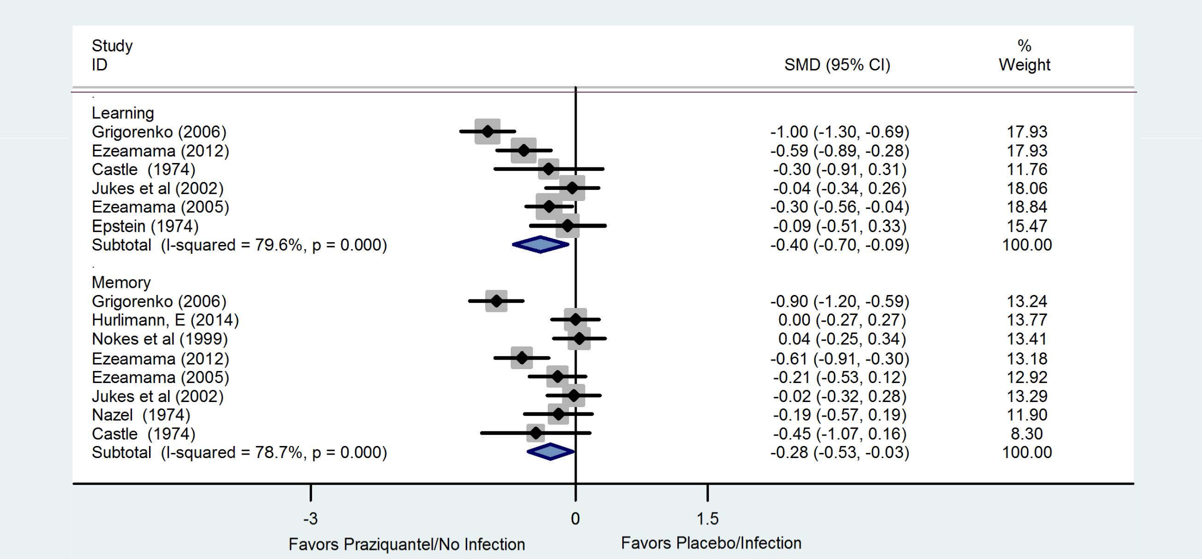

An estimated 800 million persons in tropical and subtropical countries are at risk of infection by one of three main human *Schistosoma* parasites - *S. mansoni*, *S. haematobium*, and *S. japonicum*.

Findings

- *Schistosoma* infection was associated with small-to-moderate deficits in psychometric tests of learning and memory.
- Infection was also associated with lower school attendance and scholastic achievement. Average effects on scholastic achievement were substantially larger for infection with *S. haematobium* than with *S. mansoni* infection.
- Deficits in learning and memory were clear both in analysis of observational studies and in longitudinal studies. Deficits related to educational achievement appeared robust among observational studies. They were less robust in the longitudinal studies, and risk of study bias was of potential concern.

The small-to-moderate deficits we observed at the individual level may amount to large and important differences in population achievement at the community level. It is not currently possible to estimate what the lifetime impact might be for individuals, as relatively small decrements in cognition or educational attainment in childhood may have larger impact on personal performance in later adult life.

Implications for school-aged children

Schistosoma infection often occurs in the context of malnutrition, coincident parasitic infections, and extreme poverty. Given the impacts of *Schistosoma* infection, increasing efforts to prevent or eliminate this disease are critical. If that is not yet possible, ensuring infected children receive treatment is important for their health and well-being. We hope that future studies of early childhood interventions, including treatment of schistosomiasis, will clarify to what extent the deficits we observed are reversible.

Implications for pre-school-aged children

Children from endemic areas are often infected by two years of age and remain chronically infected throughout their school-age years. These children may therefore suffer cumulative damage to their health and functioning that is currently not reflected in most short-term study outcomes. At present, there is no specific guidance for anti-*Schistosoma* drug treatment of pre-school children, partly because of the lack of a child-friendly pediatric formulation.

Given that we observed an adverse impact of *Schistosoma* infection on cognitive and educational domains in school-aged children, it is likely that the impact on younger children is at least as large or larger. Future longer-term studies should evaluate the impact of infection on pre-school children. These would provide important information for guiding decisions about preventive chemotherapy for and ensuring regular treatment of infected pre-school children.

[1]Ezeamama AE, Bustinduy AL, Nkwata AK, Martinez L, Pabalan N, Boivin MJ, et al. (2018) Cognitive deficits and educational loss in children with schistosome infection—A systematic review and meta-analysis. PLoS Negl Trop Dis 12(1): e0005524. https://doi.org/10.1371/journal.pntd.0005524

SCORE Rapid Answers Project (RAP):

The Decline in Infection-Related Morbidities Following Drug-Mediated Reduction in the Intensity of *Schistosoma* Infection

Background: Since 1984, WHO has endorsed drug treatment to reduce *Schistosoma* infection and its consequent morbidity. Cross-sectional studies suggest pre-treatment infection intensity correlates with risk for *Schistosoma*-related pathology. However, other evidence suggests that even if drug treatment reduces intensity, morbidity may not be reversed because some morbidities occur at all levels of infection, and some reflect permanent tissue damage. The aim of this project was to systematically review evidence on the impact of drug-based control on schistosomiasis-related morbidities, and to develop a quantitative estimate of this impact.[1]

Question: Does treatment of *Schistosoma* infection translate into reduced odds of infection-related morbidity? If so, by how much?

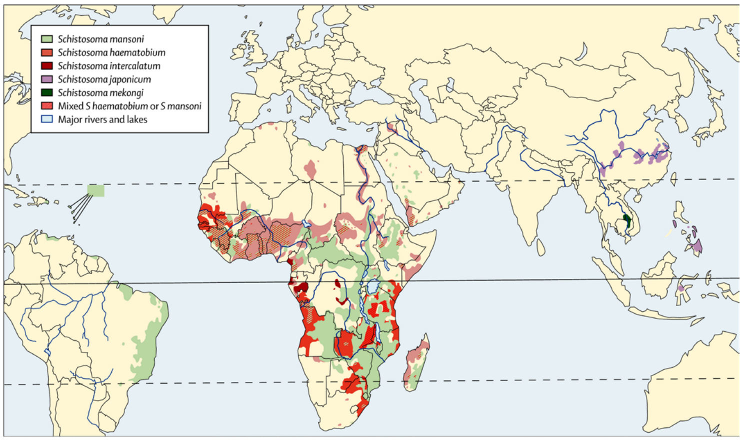

From: Colley, DG et al. (2014) Human Schistosomiasis, Lancet 383:2253-64.

Morbidities that Could be Evaluated by Meta-analysis

For <u>intestinal schistosomiasis</u> caused by *S. mansoni* or *S. japonicum*: blood in stool, diarrhea, periportal fibrosis, portal vein dilation, splenomegaly, right-sided hepatomegaly, left-sided hepatomegaly, anemia

For <u>urogenital schistosomiasis</u> caused by *S. haematobium*: upper urinary tract lesions, bladder lesions, proteinuria, hematuria, anemia

The Problem: Schistosomiasis is the disease caused by infection with *Schistosoma* parasitic flukes. Infection can cause anemia, diarrhea, abdominal pain, difficulties with learning, and decreased physical fitness. Depending on the infecting species, chronic *Schistosoma* infection can cause a variety of pathologies. *S. mansoni* and *S. japonicum* can cause liver and spleen enlargement, fibrosis and hypertension of the portal vein of the liver, and in some cases, death due to gastrointestinal bleeding. *S. haematobium* can cause bladder ulceration and kidney blockage, bladder cancer, and genital lesions that lead to fertility problems in both women and men.

Approach: In our study, we quantified the reductions in prevalence of infection-related morbidities among populations with *Schistosoma* infection, as achieved by giving one or more drug treatments. We systematically reviewed 71 available reports of *Schistosoma*-related morbidity reduction and conducted a meta-analysis of the available data to quantify the odds of persisting morbidity after treatment in relation to the egg reduction rate, ERR, a measure of how much egg counts change from pre- to post-treatment. A higher ERR indicates a greater impact on infection intensity with drug treatment.

Worldwide, schistosomiasis control is a constant challenge for public health services in endemic regions, mainly due to difficulties in preventing frequent reinfection during childhood and early adulthood. Chronic or recurrent infections lead to progressive inflammatory damage from parasite eggs that remain trapped in human tissues.

Key Findings

Our meta-regression indicates that post-treatment reductions in egg burden are significantly correlated with decreased morbidity. In particular, larger ERRs, which indicate acute reductions in worm burden, are associated with reversal of most acute pathology. More advanced chronic pathologies appear less responsive to single rounds of treatment, even with adequate ERRs, multiple rounds of treatment may be necessary to improve those outcomes. Factors affecting the magnitude of morbidity reductions include *Schistosoma* species, population studied, age and infection status of study participants, and how long after treatment follow-up occurred.

Schistosomiasis Consortium for Operational Research and Evaluation
CTEGD, University of Georgia, Athens, GA 30602
phone: 706-542-3334 • fax: 706-542-3582 • http://score.uga.edu

Morbidity Reduction

I. Examples of Treatment Impact

Relative reduction in odds of diseases after treatment of *S. haematobium*

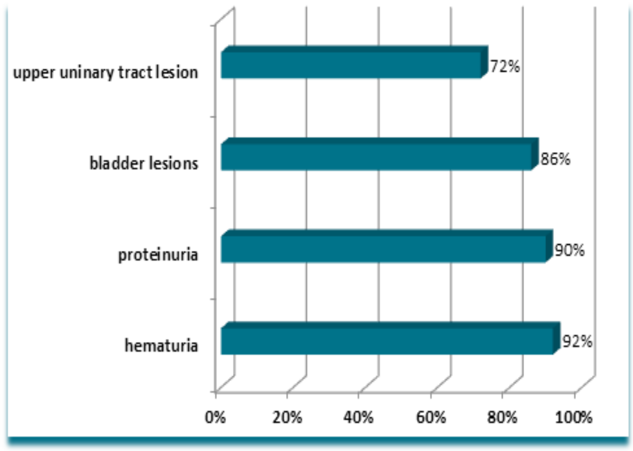

Relative reduction in odds of diseases after treatment of *S. mansoni or S. japonicum*

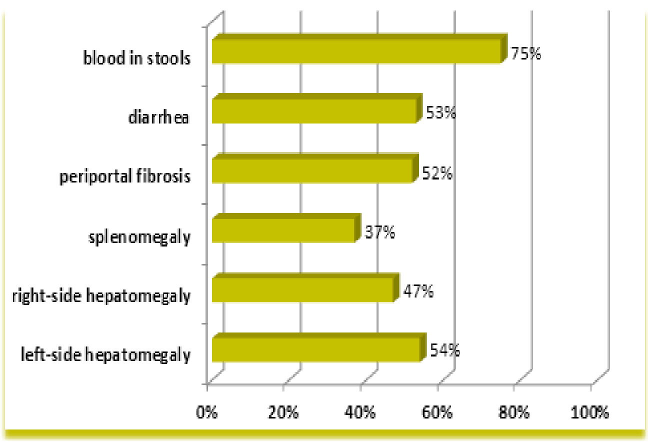

There were no consistent changes in portal dilation or blood hemoglobin levels.

II. Examples of Meta-Regressions Showing Relations Between ERRs and Odds of Diseases Post-Treatment

The graphs below plot the relative odds of disease post-treatment against ERRs. The higher the ERR, the greater the impact of treatment on infection intensity. These plots show that higher ERRs are associated with lower odds for post-treatment disease. Meta-regression lines (center black lines) and their 95% confidence limits (upper and lower blue lines) are shown for urinary tract bleeding when subjects had *S. haematobium* infections (left panel), and for periportal fibrosis of the liver when subjects had *S. mansoni* or *S. japonicum* infections (right panel).

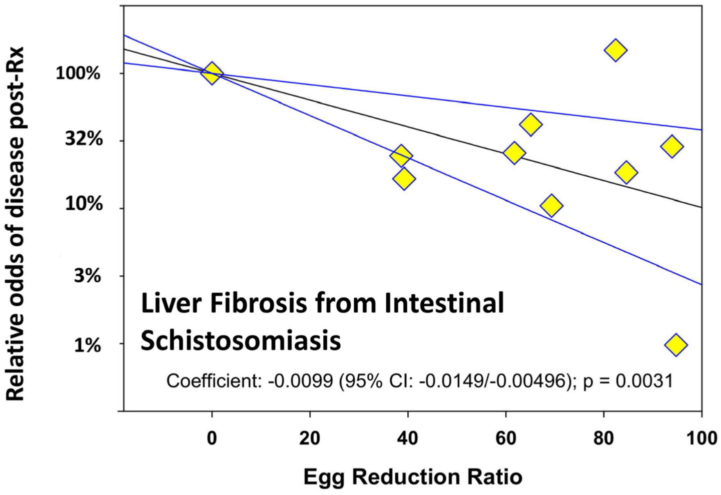

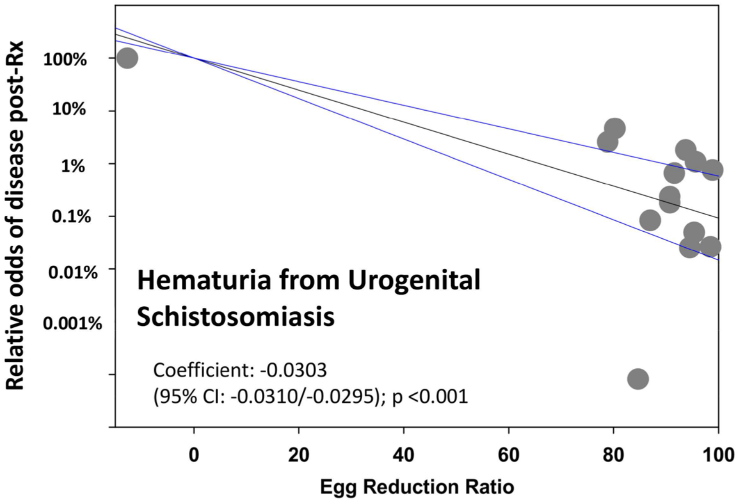

Implications

Our study shows that oral drug treatment reduces disease burden and supports continued efforts to reach populations at risk. Reducing all morbidity may require providing repeated treatment for people at risk for chronic and more intense infection. Because the reduction in egg output is significantly correlated with decreased morbidity, our estimates of the post-treatment odds of morbidity can be used to predict diminution in disease burden after successful program implementation. Nevertheless, our study was limited by gaps in the literature; additional well-designed and well-reported cohort studies are needed to strengthen the evidence base related to treatment impact on *Schistosoma* morbidity control.

[1]Andrade G, Bertsch DJ, Gazzinelli A, King CH (2017) Decline in infection-related morbidities following drug-mediated reductions in the intensity of *Schistosoma* infection: A systematic review and meta-analysis. PLoS Negl Trop Dis 11(2):e0005372. doi:10.1371/journal.pntd.0005372
